# American Medical Association

**Published:** 1879-05-20

**Authors:** 


### *AMERICAN MEDICAL ASSOCIATION.*

The above body, composed of eminent and representative men of the Profession from every State in the Union, convened in Atlanta, Georgia, May the 6th inst., at 11 o'clock a.m., in the Opera House.

As our space will not permit the publication of the minutes in detail we will attempt only a synopsis of the more important measures.

The General Sessions were held in the Opera House daily, at from 9½ to 1 o'clock, and the several Sections met at different halls, at 3 o'clock p.m.

The Association was called to order by the President, Dr. Theophilus Parvin, of Indianapolis.

Dr. J. P. Logan, of our city, Chairman of the Committee of Arrangements, made the address of welcome in warm and appropriate language.

After the registration of members, a very able, interesting and eloquent address was delivered by Dr. Parvin, the President. The address made a profound impression and is regarded among the finest, most erudite and brilliant efforts ever made on a similar occasion.

Among the measures first introduced were resolutions by Dr. Seguin, of New York, favoring the general adoption of the Metric System in the Uuited States. The resolutions were adopted.

On the second day of the General Session, a communicatiou against the removal of the duty on quinine was presented, which occasioned much interest and resulted in decided opposition—the proposition being tabled, and a large majority voting for a request to Congress to abolish the duty upon quinine.

A paper by Dr. S. Billups, of Washington, on "Sanitary matters" was read by Dr. Woodward. The advantages and necessity of national quarantine were fully set forth.

An amendment to the Code of Ethics against teaching students of irregular systems of medicine was discussed. Professor E. S. Dunster spoke in opposition to the amendment. His remarks were forcible, logical and impressive.

After considerable discussion, a motion to lay the amendment on the table until next year prevailed.

An able paper on "Pus" was read by Dr. Moses Gunn, of Chicago.

On the third day of the General Session, the Committee on Nominations reported as follows, which was adopted :

### REPORT ON NOMINATIONS.

President, Dr. LEWIS A. SAYRE, of New York.

Vice Presidents ;

- 1st. Dr. R. Beverley Cole, of California.

- 2d. Dr. E. M. Hunt, of New Orleans.

- 3d. Dr. H. O. Marcy, of Massachussetts.

- 4th. Dr. F. P. Porcher, of South Carolina.

Treasurer, Dr. R. J. Dunglison, of Pennsylvania.

Librarian, Dr. Wm. Lee, of the District of Columbia.

Committee on Library, Dr. Johnson Eliot, D. C.

Assistant Secretary, Dr. Walter Gillette, New York.

Next place of meeting, New York ; time, first Tuesday in June 1880.

Committee of Arrangements in New York : Drs. L. O. Vanderpool, Stephen Smith, W. M. Polk, Robert Weir, Charles I. Paidee, A.A. Smith, T.F. Sabine, J. Hutchison (Brooklyn), H. M. Burton, of Troy. and Dr. Parker, of Poughkeepsie.

Committee on Prize Essays, Dr. Austin Flint, Chairman.

Chairmen of Sections :
- *Practice, etc.*, Dr. S. Lynch, of Md.
- *Obstetrics, etc.*, Dr. Albert Smite, f Pa.
- *Surgery, etc.*, Dr W. T. Briggs, of Tenn.
- *State Medicine, etc.*, Dr. J. F. Hibbard, of Indiana.
- *Ophthalmology, etc.*, Dr. Booling A. Pope, of La.

Committee on Necrology, Dr. J. M. Toner, of D. C., Chairman.

Committee to Represent the Association Abroad, Drs. Seguin, Yandell, Costa, Gunn, Turnbull, Warren and Hodgson.

Committee on Public Hygiene and its Regulation by Congress, Drs. Pratt, Davis, Garcelon, Gross and Bell.

The prize for Essay the past year was awarded to Dr. McLane Hamilton, of New York. Subject: "Primary and Secondary Degeneration of the Lateral Columns of the Spinal Cord." Prize awarded, $100.

### THE SECTIONS.

In the Section on Practice of Medicine, there were presented at the several meetings the following papers :

A paper on practice, "Materia Medica and Philosophy," by Dr. Rochester, of New York.

"Clinical and Meteorological Records," by Dr. Davis, of Chicago.

"Experience of Consumptives, etc., in Colorado," by Dr. Dennison, of Colorado.

"Water in the Treatment of Diseases," by Dr. L. D. Bulkley, of New York.

Dr. Rochester's paper in favor of quarantine against yellow fever gave rise to a discussion exhibiting much difference of opinion as to the question of importation of the disease. A majority favored a national system of quarantine.

A paper on "Veratrum Viride," by Dr. G. F. Cooper, of Ga.

"Plastic Bronchitis, with Cases," by Dr. Glasgow, of Mo.

"Inflammation of the Hair Follicles of the Beard," by Dr. Shoemaker, of Philadelphia.

These several papers were discussed by the members present.

At the Section of Obstetrics and Diseases of Women and Children, there was presented a paper on "Tubo-Ovarian Pregnancy-Operation," by Dr. Robert Battey, of Georgia.

"Electrolisis of Fibroid ," by Dr. E. Cutter, of Massachusetts.
"Dysmenorrhœa," by Dr. W. H. Byford, of Illinois
Dr. Dunster, of Michigan, made some remarks in which facts were mentioned tending to show that the method of constipating the bowels after the operation for lascerated perineum was an error.
The new "Gynecological Table" devised by Dr. Chadwick. of Boston, was presented and explained by Dr Marcy.
A paper on "Treatment of Uterine Displacements by the Stem-Pessary," by Dr, E. Cutter, of Mass.
A new "Instrument for Operation for Vesico-Vaginal Fistula, wiih Cases, etc.." by E. A. Turnipseed, of S.C. It embraces a number of different instruments combined in one, displaying great mechanical ingenuity.
Drawings, with "Remarks on Lascerated Perineum" were presented by Dr. Pollen, of New York. He described his operation of the "Amputation of the Cervix," which he styled "Vagino-Cervisplasty."
Dr. Marcy addressed the Section on Dr. Garland's "Plan of Treating Prolapse of the Cord by Rotating the Body of the Fœtus in the Uterus, and thus Winding the Cord upon the Body."

At the Section on Surgery, was read a paper on "Deformities of Face and Hands, occasioned by Cicatrical Contraction from Burns, with Cases."
A paper on "Aspiration of Knee Joint, with Cases," by Dr. H. O. Marcy, of Mass.
The following papers were read by Dr. Turnipseed, of S. C. :
"New Surgical Needle, Curved and Spring Clamp at Points."
"New Apparatus for Treating Fracture of the Clavicle, with Cases."
"New Method of Reducing Dislocation of Elbow Joint, with Cases.
A voluntary paper on a case of "Chronic Dislocation of Hip Joint" was read by Dr. Matham, of Kansas.
Specimens of Calculi were exhibited by Dr. Dawson, of Ohio.
A paper was read by Dr. Lewis A. Sayre, of New York, on the "Treatment of Pott's Disease by Suspension and Retention of the Improved Position by the plaster of Paris Bandage." The Doctor illustrated his plan by the application of the bandage to a patient in the presence of the Association.
A paper was read on "Amputations by Open Cone-Shaped Method," by Dr. J. E. Link, of Indiana.
A paper on "Urinary Calculus, with 46 Cases," by Dr. H. F. Campbell, of Georgia.
A paper on "Ecraseur for Removal of Uterine Tumors," invented by the author, Dr. Wm. Scott.
"Treatment of Hemorrhoidal Tumors by Carbolic Acid Injection," by Dr. J. Weist, of Indiana.
"Nature of Gonorrhœa," by Dr. Maddox, of Md.
"Perityphitic Abscess Opening into the Bladder and Rectum," by Dr. Rochester.
A new instrument for the "Administration of Anæsthetics," by Dr. A. M. Pollock, of Penn.

In the Section embracing Medical Jurisprudence, Chemistry, Psycology, State Medicine and Hygiene, an elaborate and interesting paper was r ad on "State Medicine and State Medical Societies," by Dr. S. E. Chille, of New Orleans.
Dr. E. Seguin, of New York, read a scientific paper, in which it was held that the education of the intellect of idiots must begin by the education of the senses. A case in point was related.
A paper on "New Principles of Protective Sanitation in Relation to Public Hygiene," by Dr. H. R. Storer, of R. I.
Resolutions were adopted asking all physicians to aid the census agents in procuring statistics of mortality, by keeping a record of all their cases from the first of January, and urging the organization of State and County Societies.
A paper on "Treatment of Small Pox in the Initial Stages of the Fever," by Dr. A. S. Payne, of Va.

In the Department of Ophthalmology, a paper was read on "Ivory Bony Tumor of the Socket of the Eye."
Also a paper on "Impairment of Sight from Excessive Doses of Quinine," by Dr. Voorhees, of Memphis.
Papers on "Cataract" were read by Drs. Pope, Calhoun and Knapp.
A paper on the "Operative Cure of Cystoid Cicat ix Following Operations for Cataract and Glaucoma," by Dr. Reynolds, of Louisville.

After passing the usual resolutions of thanks, and feeling and appropriate addresses by the outgoing and incoming Presidents, the Association adjourned.
We have only to add that the occasion was a great one for Atlanta, and one to which our citizens will ever recur with recollections of pride and pleasure. W.

